# Metabolic Syndrome in people treated with Antipsychotics (RISKMet): A multimethod study protocol investigating genetic, behavioural, and environmental risk factors

**DOI:** 10.1371/journal.pone.0298161

**Published:** 2024-05-01

**Authors:** Giovanni de Girolamo, Caterina La Cascia, Paolo Emidio Macchia, Maria Nobile, Stefano Calza, Laura Camillo, Maddalena Mauri, Marco Pozzi, Giada Tripoli, Claudia Vetrani, Elisa Caselani, Marta Magno

**Affiliations:** 1 Unit of Epidemiological and Evaluation Psychiatry, IRCCS Istituto Centro San Giovanni di Dio Fatebenefratelli, Brescia, Italy; 2 Department of Biomedicine, Neuroscience, and Advanced Diagnostics, Section of Psychiatry, University of Palermo, Palermo, Italy; 3 Department of Clinical Medicine and Surgery, Endocrinology Unit, University “Federico II”, Naples, Italy; 4 AOU Policlinico “Federico II”, Naples, Italy; 5 Education for Health and Sustainable Development, University of Naples “Federico II”, Naples, Italy; 6 Child and Adolescent Psychiatry Unit, Scientific Institute IRCCS Eugenio Medea, Lecco, Italy; 7 Department of Molecular and Translational Medicine, University of Brescia, Brescia, Italy; 8 Dipartimento di Scienze Umanistiche, Università Telematica Pegaso, Centro Direzionale, Napoli, Italy; Public Library of Science, UNITED STATES

## Abstract

**Introduction:**

The RISKMet project aims to: (1) identify risk factors for metabolic syndrome (MetS) by comparing patients with and without MetS; (2) characterise patients treated with second-generation antipsychotics (SGAs) about MetS diagnosis; (3) study behavioural patterns, including physical activity (PA) and dietary habits, in patients and healthy individuals using a prospective cohort design.

**Method:**

The RISKMet project investigates MetS in individuals treated with SGAs, focusing on both adult and paediatric populations. The study utilizes a case-control design to examine potential risk factors for MetS, categorizing participants as MetS+ considered as “Cases” and MetS- considered as “Controls” matched by sex and age. The evaluation of factors such as MetS, lifestyle habits, and environmental influences is conducted at two time points, T0 and T3, after 3 months. Subsequently, the project aims to assess body parameters, including physical examinations, and blood, and stool sample collection, to evaluate metabolic markers and the impact of SGAs. The analysis includes pharmacological treatment data and genetic variability. Behavioural markers related to lifestyle, eating behaviour, PA, and mood are assessed at both T0 and T3 using interviews, accelerometers, and a mobile app. The study aims to improve mental and physical well-being in SGA-treated individuals, establish a biobank for MetS research, build an evidence base for physical health programs, and develop preventive strategies for SGA-related comorbidities.

**Conclusions:**

This project innovates MetS monitoring in psychiatry by using intensive digital phenotyping, identifying biochemical markers, assessing familial risks, and including genetically similar healthy controls.

**Study registration number:**

ISRCTN18419418 at www.isrctn.com.

## Introduction

Many individuals who suffer from mental disorders in both childhood and adulthood [1] and are undergoing antipsychotic (AP) treatment are at significant risk of physical comorbidities. Second-generation antipsychotics (SGAs) are commonly prescribed to manage symptoms primarily linked with schizophrenia spectrum disorders (SSD) [2, 3], bipolar disorder (BD) [4] and other mental health conditions in paediatric populations, such as disruptive behaviour disorders and irritability or aggression in the context of neurodevelopmental disorders [5].

The rise in the number of associated medical conditions in these patients can be attributed to several underlying mechanisms: (a) pathogenetic and biological factors (e.g., metabolic or immune system disorders, genetic susceptibility); (b) unhealthy behaviours, including poor diet, smoking, sleep disturbances and low levels of physical activity; (c) low utilisation of healthcare services, often due to stigma; and (d) negative consequences of common medications (e.g., side effects). APs, lithium and other medications, as well as a high prevalence of concurrent mental health disorders, including substance use disorders are commonly associated with comorbidities [6, 7]. The most frequent medical comorbidities in psychiatric patients [8, 9] include type-2 diabetes, cardiovascular disease (CVD), and endocrine disorders, such as metabolic syndrome (MetS) [10]. MetS is indicative of a preclinical state characterised by a cluster of risk factors for CVD and type-2 diabetes mellitus, which tend to cooccur in individuals with MetS [11]. The criteria for diagnosing MetS in both adult and paediatric populations can be found in Table 1. The prevalence of MetS is markedly high among patients with SSD, BD and NDD [12]: notably, many of those patients underwent prolonged treatment with APs, often in combination.

**Table 1:** Diagnostic criteria for Mets in adults and paediatric populations.

**ADULT** *	**VALUES**
Waist circumference	>102 cm (M); >88 cm (F)
Fasting glucose	>100 mg/dL
Blood pressure	>130/85 mmHg
Triglycerides	>150 mg/dL
HDL cholesterol	<40 mg/dL (M); <50 mg/dL (F)
PAEDIATRIC POPULATION**	**VALUES**
Waist circumference	>90th percentile
Fasting glucose	>100 mg/dL (or known type 2 diabetes)
Blood pressure	>90th percentile for age, sex, height
Triglycerides	>150 mg/dL
HDL cholesterol	<40 mg/dL

* MetS+ in individuals meeting at least three criteria.

**MetS+ in paediatric subjects with central obesity plus two or more additional abnormalities.

### Physiopathological link between APs and MetS

**Fig 1:**
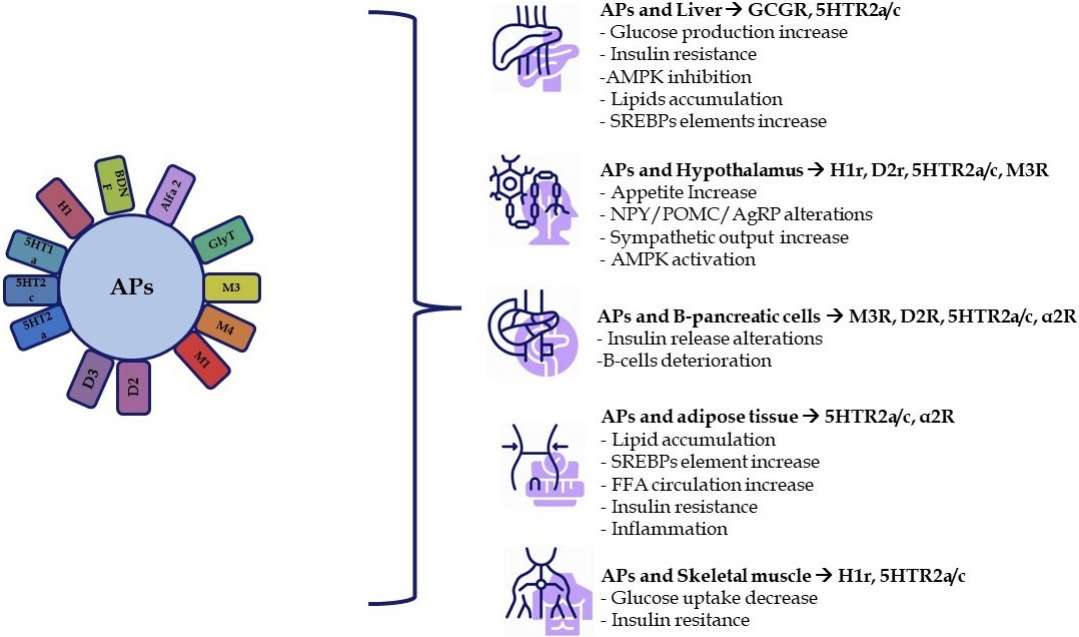
The development of MetS caused by APs is a result of their multifaceted impact on both the central nervous system (CNS) and peripheral organs. Within the CNS, the primary focal point is the hypothalamus, whereas, in the periphery, APs affect various organs, including the liver, pancreatic β-cells, adipose tissue, and skeletal muscle, all contributing to the onset of MetS. This disruption in glucose and lipid homeostasis is primarily attributed to APs’ interference with the activity of various G protein-coupled receptors (GPCRs) present in these regions [16, 18].

AP drug treatment is known to dysregulate glycaemic and lipid metabolism, thereby contributing to the development of MetS in patients [13]. It was previously hypothesised that a patient’s genetic and environmental background may entirely contribute to MetS [14] however, it is now clear that the mechanisms underpinning MetS are multifactorial and complex. AP treatment has been linked to weight gain, impaired glucose metabolism, worsening of preexisting type-1 and type-2 diabetes, new-onset type-2 diabetes mellitus, and diabetic ketoacidosis, which are all known risk factors for the onset of MetS [15]. The complex molecular and cellular mechanisms driving these metabolic changes are diverse and encompass nearly all organs involved in metabolic processes [16]. Numerous receptors and proteins are affected by APs, which also influence the activity of neuromodulators and hormones. The low affinity for D2 receptors, the participation of serotonin receptors, and consequently, the ratio of 5ht2a/D2 receptor involvement associated with AP use are deemed the primary characteristics ubiquitous to all APs, but they do not entirely elucidate the variations in metabolic outcomes associated with the use of different APs [17]. Many investigations underscore the significance of other receptors in that relationship. Not only are there receptors linked to dopamine and serotonin that are involved in that relationship, but G-protein coupled receptors (GPCRs) are also activated [18]. GPCRs comprise glucagon (GCG) receptors, glucagon-like peptide-1 (GLP-1) receptors, gastric inhibitory polypeptide (GIP) receptors, muscarinic receptors, free fatty acid (FFA) receptors, adrenergic receptors, dopamine receptors, serotonin receptors, and ghrelin receptors. GPCRs, which are targeted by APs, play a crucial role in the pathophysiological links between APs and MetS because they regulate energetic metabolism in almost all peripheral tissues. In the central nervous system (CNS), APs disrupt hypothalamic activities by targeting GPCRs, thereby modifying descending and ascending autonomic control [16]. Furthermore, adverse metabolic effects of APs have been described that occur directly at the intracellular level due to the disruption of cholesterol and lipid metabolism and trafficking, lysosomal function and autophagy [19]. The effects of APs on the central nervous system and peripheral organs and the receptor-dependent mechanisms leading to MetS are shown in Fig 1.

### Biomarkers in MetS

The clinical phenotype of MetS contributes to the development of a pro-inflammatory state defined by an increase in oxidative stress and subclinical vascular inflammation. Based on the MetS phenotype, biomarkers can be categorized into four macrogroups, namely, dyslipidaemia, oxidative stress, inflammation, and cardiometabolic markers. These groups are closely related to each other and are associated with an increased risk of developing further pathologies [20]. Dyslipidaemia comprises a variety of lipid irregularities, including disturbances in the composition, processing, and functional role of atherogenic lipoproteins and protective HDL-C, indicated by both qualitative and quantitative indicators. These abnormalities comprise increased levels of chylomicrons, VLDL, LDL, and apo-B-carrying lipoproteins, increased triglycerides, and decreased concentrations of HDL cholesterol. Oxidative stress plays a role in cellular dysfunction and an increase in manifestations related to MetS, such as atherosclerosis and hypertension. According to Jin et al. (2010), patients with MetS have higher levels of GGT, oxidized LDL, ferritin, and uric acid [21]. MetS exhibits a stable and slight inflammatory level, which facilitates the generation of further complications [20]. Classical metabolic biomarkers, including insulin and glycated haemoglobin (HbA1c), as well as adipokines, such as adiponectin and leptin, are some of the cardiometabolic markers. These two biomarker subgroups reciprocally impact each other, leading to higher rates of both cardiovascular disease and all-cause mortality [21].

### Physical activity and sleep quality in people with mental disorders and measurement tools

Evidence suggests [22] that MetS is positively linked with physical inactivity bouts, while good sleep patterns are associated with better cardiometabolic health in cross-sectional and prospective studies [23]. Furthermore, patients treated with APs consistently report less physical activity (PA) and higher levels of sedentary behaviour [24, 25] as well as poorer sleep quality and greater variability in sleep duration than control populations. Sleep disturbances were found to contribute to CVD risk, independent of major confounding factors, in different clinical populations with mental disorders [26]. In recent years, advancements in wearable biosensors (accelerometers) have significantly enhanced the assessment of daily real-life PA and sleep-wake rhythms [27]. Accelerometers are readily available commercial devices [28] and serve as a pivotal tool in this field of research.

### Experience sampling method in mental health

Time-use surveys are frequently conducted using semistructured interviews to retrospectively gather information about daily activities, making them a valuable tool for studying time distribution and trends among various groups. However, when evaluating time use in individuals with mental disorders, there is a risk of bias due to memory impairments. These individuals may struggle to accurately recall and report their daily activities, potentially leading to inaccuracies in traditional survey methods. To address this issue, recent research has explored alternative approaches, such as the experience sampling method (ESM). ESM enables real-time, ecologically valid self-reports of an individual’s activities, emotions, and social interactions in everyday life, reducing the reliance on memory and providing a more accurate overview of time allocation [29]. This methodology is particularly useful for analysing person-environment interactions and can assess individuals in various settings, including those with cognitive impairments. When complemented with real-time metrics such as accelerometers, ESM enhances the assessment of behavioural determinants. The objective of this project is to enhance the mental and physical well-being of individuals undergoing SGA treatment, create a biobank for MetS research, establish a foundation of evidence for physical health programs, and formulate preventive strategies for comorbidities associated with SGA use.

### Objectives

Studying MetS in patients treated with APs, including not only a clinical and biological but also a behavioural investigation, allows us to identify high-risk populations and possibly prevent the progression of some of the major causes of morbidity and mortality in clinical populations treated with these compounds. The RISKMet project has three objectives, each with distinct research phases.

1. The first objective is to ascertain risk factors for MetS in patients treated with APs, particularly SGAs. The study will utilize a case‒control design, with two subject groups recruited from both adult and paediatric populations: patients diagnosed with MetS (cases, MetS+) and patients without MetS (controls, MetS-), who will be matched for sex, age group, disease severity, using Clinical Global Impression (CGI) [30], diagnosis and AP treatment. To identify risk factors for MetS, a thorough assessment of familiarity with MetS will be carried out, together with an assessment of psychological and functional risk factors (e.g., behavioural characteristics, disability, quality of life, diet, sleep quality).
2. The second objective is to thoroughly assess the present health status of patients who have been given SGAs and either have or do not have symptoms of MetS.
3. The third objective is to prospectively investigate behavioural patterns linked to MetS, including PA, sleep, and dietary habits. PA and sleep will be tracked with a wrist-worn accelerometer, while eating behaviours (such as daily caloric intake, binge eating episodes, night-time eating, cravings, fast food consumption, and satiety) will be monitored through a mobile-based ESM.

## Materials and methods

### Design and setting of the study

**Fig 2:**
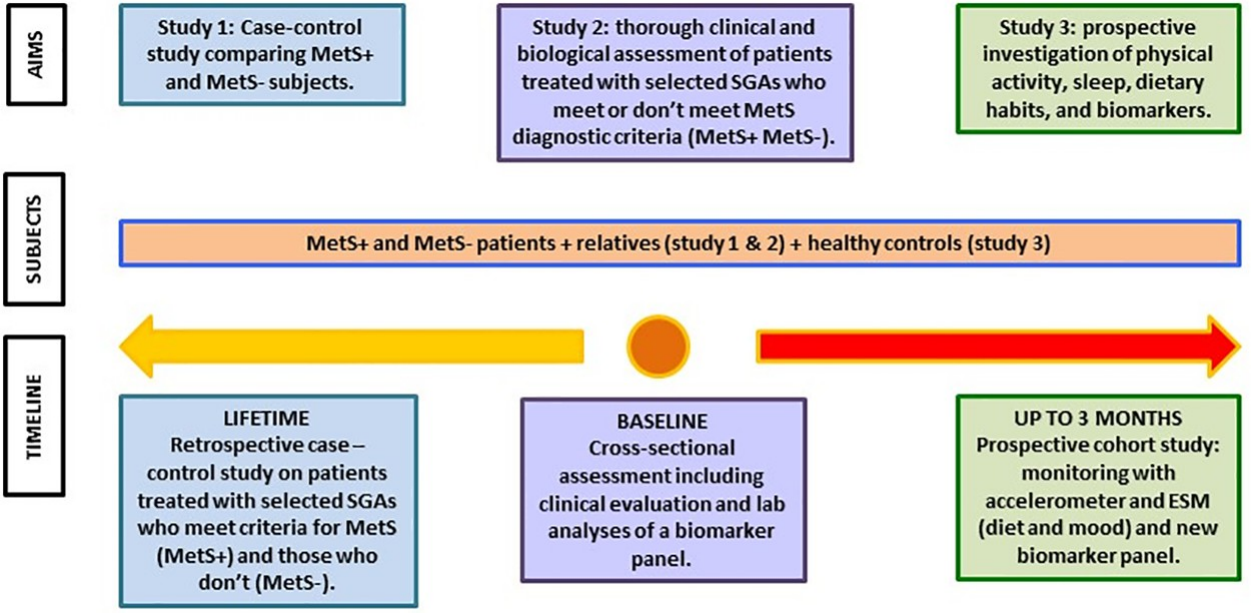
Overall design of RISKMet.

As stated before, the project includes three different phases, with different study designs (Fig 2): the first phase has a case‒control design, the second phase involves a detailed cross-sectional assessment, and the third phase uses a cohort design, with two evaluation points: the first and second phases will occur during the enrolment of study subjects (T0), while the third phase will take place over a 3-month follow-up (T3). The whole project will have a duration of two years.

The RISKMet study is an Italian multicentre investigation. The adult cohort, consisting entirely of outpatients, will be recruited at two sites: IRCCS Fatebenefratelli in Brescia and ‘Giaccone’ Hospital in Palermo. Meanwhile, the paediatric population sample will be recruited from IRCCS Medea in Bosisio Parini (LC), Italy, focusing on outpatient participants. The biological samples will undergo analysis at ‘Federico II’ Hospital in Naples. Further information about the participating sites is provided in S1 Table. This study was approved by the Ethics Committees of all participating centers. It was approved by the Comitato Etico IRCCS San Giovanni di Dio—FBF (approval no. 36–2023), by the Comitato Etico IRCCS Eugenio Medea—sezione scientifica dell’Associazione “La Nostra Famiglia” (approval no. 26/23) and by the Comitato Etico Palermo 1 (approval no. 06/2023).

### Study 1: Characteristics of the participants

Adult MetS+ patients aged 18–65 years will include individuals diagnosed with SSD, BD I, or BD II according to DSM-5 criteria who do not have a serious cognitive impairment ascertained with the Montreal Cognitive Assessment (MoCA) [31]. Paediatric MetS+ patients aged 6–17 years will be individuals diagnosed with NDDs such as autism spectrum disorder, intellectual disability, and tic disorders according to DSM-5 criteria. All participants classified as MetS+ will be individuals who have received treatment with selected SGAs (e.g., olanzapine, risperidone or aripiprazole) for at least one year (and at least six months in the last year) and have fully met diagnostic criteria for MetS during the previous year. The three SGAs were selected because, based on pharmacoepidemiologic data of Italian registries of drug prescriptions, they are the three most widely used APs, both in adults and in children/adolescents. Table 1 outlines the diagnostic criteria for MetS in both adult and paediatric populations. Participants classified as MetS- will be patients of the same sex and age group with the same diagnosis who have received treatment with the same compounds for the same length of time but do not meet the diagnostic criteria for MetS. All eligible MetS+ individuals meeting the inclusion criteria will be chosen randomly from individuals being referred to the recruitment centres, while MeS- patients will be randomly chosen based on one-to-one matching with MetS+ subjects. Refusals to participate in the research study will be duly recorded. To achieve random selection from the pool of individuals treated with APs in the previous year, we will assign unique integer identifiers, and a subset corresponding to the desired sample size will be extracted based on a list of random numbers generated using a random block algorithm. In addition, one parent of any adult patient will also be recruited. Detailed data on the medical history, treatment and risk factors for the parent (if alive) will be collected. In study 1, a parent shall also be selected for MetS+ and MetS- paediatric patients. To investigate the familial genetic predisposition to MetS, thorough clinical evaluations, including in-depth interviews, physical examinations (refer to S2 Table), and laboratory tests, will be conducted on parents of paediatric patients. Table 2 details the inclusion and exclusion criteria, while Table 3 displays the sample and its composition.

**Table 2:** Inclusion and exclusion criteria.

ADULT POPULATION	
INCLUSION CRITERIA	EXCLUSION CRITERIA
Age between 18–65 years	Plan to relocate in the subsequent year
Primary diagnosis of SSD, or BD I or BD II	Severe substance/alcohol misuse
Treated with aripiprazole, olanzapine or risperidone for at least six months in the last year.	Severe cognitive impairment as ascertained with the MOCA
**PAEDIATRIC POPULATION**	
Age between 6–17 years	Plan to relocate in the subsequent year
Primary diagnosis of NND (including autism-spectrum disorders, intellectual disability and tic disorders)	Severe substance/alcohol misuse
Treated with aripiprazole, olanzapine or risperidone for at least six months in the last year.	Severe cognitive impairment (QIT < 40)

**Table 3:** Stratification variables.

	GROUP	VARIABLES
**Age**	1	6–17 years
	2	18–45 years
	3	46–65 years
**Sex**	1	Male
	2	Female
**Disorder severity, as classified with CGI**	1	Less severe
	2	More severe

### Study 1: Measures, procedures, and variables

Qualified researchers will conduct assessments and confirm diagnoses using DSM-5 checklists. Each participant’s “Patient Record” will contain clinical, sociodemographic, and treatment data for both adults and children, as well as various assessments for well-being, functional status, quality of life, comorbidity, sleep quality, and family relationships. Assessments pertinent to the developmental and well-being evaluation of paediatric patients, which are routinely included in their medical records, will be sought and recorded. The assessment instruments are listed in Table 4.

**Table 4:** Instruments used for the assessment of the patients.

ADULT PATIENTS			
Name	Description	Number of item	Scoring Range
**Brief Psychiatric Rating Scale (BPRS)** [32]	To assess the presence and severity of psychopathology.	24	1–7
**World Health Organization Disability Assessment Schedule 2.0 (WHODAS 2.0)** [33]	To measure the impact of health conditions on functioning in six life domains (cognition, mobility, self-care, getting by, activities of daily living, and participation).	12	0–100
**Specific Levels of Functioning (SLOF)** [34]	To assess psychosocial functioning in six subscales: physical functioning, self-care skills, interpersonal relationships, social acceptance, community life activities, and work skills.	43	43–215
**Elixhauser Comorbidity Index (ECI)** [35]	Method of classification of different medical comorbidities according to ICD categories.	NA	NA
**EQ5D Health Questionnaire** [36]	Self-report tool to assess the quality of life in 5 domains: mobility, self-care, usual activities, pain/discomfort, and anxiety/depression.	5	0–100
**Pittsburgh Sleep Quality Index (PSQI)** [37]	Self-report tool to assess sleep quality and structure.	19	0–21
**Short Form Health Survey (SF-36)** [38]	Self-report tool to assess the quality of life covering eight health-related domains.	36	0–100
**ALL PATIENTS**			
**Clinical Global Impressions Scale (CGI)**	Instrument to measure the severity of the disorder.	3	1–7
**World Health Organization Quality of Life (WHOQOL-BREF)** [39]	Self-administered questionnaire to assess subjective health and well-being over the previous two weeks. It covers four domains: physical health, psychological health, social relationships, and environment.	26	0–100
**PAEDIATRIC PATIENTS**			
**Developmental Behaviour Checklist (DBC-P)** [40]	Instrument to assess behavioural and emotional problems in young people aged 4–18 years with developmental and intellectual disabilities.	96	1–7
**Tanner Staging Scale** [41, 42]	Tool to assess sexual maturity in children, adolescents, and adults based on primary and secondary external sexual characteristics.	4	NA
**Development and Well-Being Assessment (DAWBA)** [43]	Package of interviews, questionnaires, and assessment techniques designed to generate ICD-10 and DSM-IV or DSM-5 psychiatric diagnoses in children aged 2–17 years; the background section is used to assess family and environmental risk factors.	NA	NA
**Child Behaviour Checklist (CBCL)** [44]	Widely used caregiver reports to identify children’s most problematic behaviours.	113	NA
**Strengths and Difficulties Questionnaire (SDQ)** [45]	Short Behavioural Screening Questionnaire assessing emotional symptoms, conduct problems, hyperactivity/disattention, peer relationship problems, and prosocial behaviour.	25	NA
**Paediatric Quality of Life Inventory (PedsQL)** [46]	A short measure of health-related quality of life in children and adolescents is to be completed by parents (proxy report) and children and adolescents (self-report).	23	0–100
**Parents Problem Checklist (PPCL)** [47]	Instrument to measure inter-parental conflict, specifically, relating to the ability of parents to cooperate and act in a coordinated manner in the executive performance of the parental task	16	6–45

NA: Not Applicable

Clinicians and mental health staff at the recruitment sites will be trained on questionnaire administration. Participants will be asked to complete the questionnaires, or they will undergo standardised interviews. A web portal will be developed to facilitate direct input of study questionnaires by clinicians with user-friendly interfaces. The online software will validate data entries to reduce the risk of potential errors. If there is no internet connection available locally, it will be possible to use an offline desktop application that has the same capabilities to store the gathered data on a computer. The data will be transferred onto the online database when an internet connection becomes accessible.

### Study 2: Characteristics of the participants

All patients enrolled in the STUDY 1 procedure will undergo the assessment outlined in Study 2.

### Study 2: Measures, procedures, and variables

Patients will undergo a structured physical examination at T0, and blood samples of both adult and paediatric patients with MetS+ and MetS- will also be collected at enrolment together with a faeces specimen for microbiota analysis. Table 5 lists the blood tests that will be executed at T0.

**Table 5:** Blood tests performed on adult and paediatric patients.

BIOMARKERS	NORMAL RANGE FOR ADULTS	NORMAL RANGE FOR PAEDIATRICS
Ab-Tg	<116 U.I/mL	< 115 U.I./mL
Ab-TPO	< 60.0 U.I./mL	< 34 U.I./mL
Adiponectin	Males <10 mcg/mL Females <14 mcg/mL	Not carried out in clinical practice
ALT	<55 U/L	Males: <41 U/L Females: <33 U/L
Apo A1	Males: 79–169 mg/dl Females: 76–214 mg/dl	Males 110–170 mg/dL Females 120–190 mg/dL
Apo B	Males: 46–174 mg/dl Females: 46–142 mg/dl	Males: 80–155 mg/dL Females: 75–150 mg/dL
AST	< 34 U/L	Males: <40 U/L Females: <32 U/L
C-Peptide	0.81–3.85 ng/mL	1.1–4.4 ng/mL
Fetuin-A Glycoprotein	Not carried out in clinical practice	Not carried out in clinical practice
Ft4	8.9–17.6 pg/mL	12–22 pmol/L
GGT	Males: < 73 U/L Females: < 38 U/L	Males: <60 U/L Females: <40 U/L
Glycemia	70–100 mg/dL	74–110 mg/dl
HbA1c	20–38 mml/mol	20–42 mmol/mol
HDL	Males: >= 40 mg/dl Females: >= 50 mg/dl	Males: >55 mg/dl low risk; 55–35 mg/dl moderate risk; <35 mg/dl high risk Females: >65 mg/dl low risk. 45–65 mg/dl moderate risk; <45 mg/dl high-risk mg/dL
Insulin	3.0–25.0 μU/mL	2.6–24.9 μU/mL
LDL	< 55 mg/dL very low-risk individuals; 55–70 mg/dL low-risk individuals; 70–100 mg/dL moderate risk individuals; 100–116 mg/dL for very high-risk individuals; > 116 mg/dL extreme risk	<100 mg/dL low-risk. 100–159 mg/dL moderate risk; >160 high risk mg/dL
Leptin	BMI 18.5–24.9: < 0.7–9.1 ng/mL; BMI 25–30: 1.3–21.2 ng/mL; BMI > 30: 3.4–32.1 ng/mL	0–10 years 2.85–47.8 mg/ml; 11–15 years 3.41–34.9 mg/ml; 16–20 years 2.57–22.4 mg/ml
Lp (a)	< 30 mg/ 100ml	<75 nmol/L
Oral Glucose Tolerance Test (OGTT)	First Value: ≤ 92 mg/dl Second Value: ≤ 180 mg/dl Third Value: ≤ 153 mg/dl	Depends on the specific test used
Protein Glycation Derangements	Not carried out in clinical practice	Not carried out in clinical practice
P-Selectin Glycoprotein	Not carried out in clinical practice	Not carried out in clinical practice
Total cholesterol	<200 mg/dL	<200 mg/dL
Triglycerides	< 150 mg/dL low risk; 150–199 mg/dL moderate risk; 200–499 mg/dL high risk; > 500 mg/dL very high risk	150 mg/dL
TSH	0.55–4.78 μI.U./mL	0.27–4.2 μI.U./mL
Vitamin D	31–150 ng/mL	20–80 pg/mL
Zinc-Alpha2-Glycoprotein	Not carried out in clinical practice	Not carried out in clinical practice
Zonulin	< 38.0 ng/mL	22.3–161.1 ng/mL

Each patient will give four blood samples: one for routine tests, one for selected biomarkers, one for RNA extraction and one for DNA analyses. The first set of analyses will be collected locally and then sent to a centralised laboratory. The second set of samples, which require specific procedures for handling and analysis, will be sent to the Naples site by a certified carrier for centralised analysis. The third sample will be collected to obtain the patient’s RNA and to analyse changes in the transcription levels of specific genes responsible for key metabolic enzymes and transcription factors. All genomic analyses will be centralised at IRCCS Medea or Naples. The third sample will be processed at the Naples site, where complementary DNA (cDNA) will be derived from the samples. This cDNA will be stored and then sent to IRCCS Medea for analysis. The analysis at IRCCS Medea will involve a panel of transcripts using the real-time RT PCR method with SYBR green probes. The transcripts probed will cover various aspects of metabolic characterization, including the biosynthesis and consumption of lipids and glucose, as well as sterol transport and reference housekeeping genes such as GAPDH and RPLP0 [19]. For each patient, a fourth sample will be collected at T0 to obtain the patient’s genomic DNA, which will be analysed using next-generation sequencing (NGS) at IRCCS Medea to characterize the component of genetic variability underlying different risks of developing MetS. For faeces collection, adult patients or parents of paediatric patients will receive a special collection kit at the time of enrolment with instructions for home collection. Faeces shall be collected as soon as possible after enrolment for T0 and stored in the freezer for subsequent analysis to perform the characterization of the microbiome.

### Study 3: Characteristics of the participants

All adult and paediatric MetS+ or MetS- patients recruited for study 1 and study 2 will undergo blood tests at T0, as described in the previous section. All patients will also participate in a PA monitoring study using a wrist accelerometer and in an ESM study, which will provide information on mood, stressors, eating behaviour and other psychosocial environmental factors. To allow a comparative analysis, a healthy control population will be recruited for this phase of the study. The recruitment of a sample of healthy controls, matched in terms of age and sex, will be conducted by distributing flyers at participating centres and utilising social networks. The healthy control group will consist of persons who have no documented record of mental illnesses or substance abuse involving alcohol or drugs and have never received treatment with antipsychotic medications. Whenever feasible, individuals who are siblings of MetS+ patients will also be enlisted as healthy controls to examine the potential horizontal family susceptibility to MetS.

### Study 3: Measures, procedures, and variables

Patients’ and controls’ PA will be monitored using a wrist accelerometer, the ActiGraph GT9X, over seven 7-day periods; during that time, dietary information will also be collected using two food diaries to be completed over 3 days (preferably Thursday, Friday and Sunday). The ActiGraph GT9X is a wearable device (combining an accelerometer with measurements of heat production and skin conductivity) that measures physiological variables and body movements over time. An algorithm converts the raw data into estimates of energy expenditure (EE) expressed in both kcal/min and metabolic equivalents (METs). The multisensor device has been shown to provide accurate results for estimating EE and time spent in different activities and to provide valid estimates of EE in the general population. The following PA parameters will be measured: (i) average PA: the average of all active minutes during the day and night; (ii) total idle time and sleep (including sleep latency, sleep efficiency and total sleep time): the average number of inactive minutes between waking up in the morning and going to bed; (iii) energy expenditure; (iv) MET rates; and (v) body position.

The first food diary should be completed as soon as possible at baseline (T0), and the second should be completed in the week before the scheduled three-month follow-up assessment (T3). The diary data will then be entered into the local software Metadieta. Finally, information on mood, stressors, eating behaviour, and various other assessments of the psychosocial environment will be collected using the ESM methodology and an app developed specifically for the project.

Every participant will undergo a 30-minute training session on the ESM procedure. They will be provided with a smartphone for installing the RISKMet app or will be given one on loan if they do not have their own. For seven days, they will carry the smartphone with them and receive a prompting auditory signal for recall, which will send a notification eight times a day at random intervals from 7 AM to 10 PM. The study participants will respond to the signal contingent report. The use of a combined approach will lead to a high level of redundancy in data collection. This means that if a specific behaviour, such as a binge eating episode, takes place but is not reported (known as a missed event-contingent recording), it could still be identified through a random signal report. The combined signal-contingent and event-contingent approach guarantees the logging of every eating episode. For instance, eight times daily, signal-contingent prompts will ask participants if any target behaviour took place since the previous prompt. If the answer is affirmative, the participants will then be asked if they completed an event-contingent report during the time of behaviour, which is preferable.

Overall, this approach presents a valuable assessment strategy for researching eating behaviours. First, behaviours are evaluated in their natural environment. Second, the method eliminates the need for lengthy retrospective self-reports. Third, it adequately characterises participants’ environmental, social, and psychological factors. Following a brief physical activity assessment training, participants will wear a multi-sensor device (ActiGraph GT9X) continuously for seven consecutive 24-hour periods. The device will be worn on the non-dominant arm and will be configured to record 60-second intervals, calculating the total activity count for every active minute throughout the day. Smartphone and actigraphy measurements will be taken at the same time (i.e., during the same week).

## Data analysis plan

Our objective is to include MetS+ patients from different sites while also incorporating a contingency plan for possible attrition. Our aim is to enrol a minimum of 25 MetS+ patients at each site. We will factor in an additional 30% to account for the anticipated dropout rate for each site, resulting in a total target enrolment of 33 patients per site. When there are several recruitment sites involved, the combined MetS+ sample size will be determined by adding up the sizes of each individual site. For example, with three recruitment sites, the overall number of patients with MetS+ will be 99. The selection of MetS- patients will follow a methodical approach to ensure that they are comparable to their MetS+ counterparts in terms of age, sex, and diagnostic category. A parallel approach to estimating the sample size will be implemented for paediatric cases at IRCCS Medea. This rigorous methodology ensures a statistically sound study that is sufficiently powered to detect significant outcomes while carefully considering potential attrition and promoting accurate comparative analysis.

### Statistical consideration: Study 1

This study aims to identify factors that contribute to MetS by contrasting MetS+ cases with MetS- controls. The target is to recruit 25 subjects for each age group, defined as 6–17, 18–45 and 46–65 years, and corresponding one-to-one matched MetS- individuals for a total of 300 participants. According to prior research, MetS is estimated to have a prevalence of approximately 40% in the study population. Using a conditional logistic model for a matched case‒control design, the chosen sample size will allow us to evaluate as significant (alpha = 0.05) an odds ratio (OR) of at least 1.98 (or 0.51), assuming a 50% probability for a binary exposure and an R2 of 10% with other covariates, with 80% power. The same setting will allow us to identify an OR of at least 1.4 (or 0.71) for a quantitative exposure with a standard deviation of 2 (PASS 2021).

### Statistical consideration: Study 2

The second study aims to conduct a cross-sectional survey to investigate the prevalence of risk factors in individuals receiving olanzapine, risperidone or aripiprazole. The analysis will commence by presenting preliminary descriptive statistics, providing an overview of vital variables and their distributions. Additionally, correlation analyses will be executed to investigate the interrelationships among the accumulated variables. These statistical approaches will facilitate the identification of patterns, trends, and plausible associations between clinical and biological parameters. There will be specific attention given to data linked with drug treatments and single-nucleotide polymorphisms (SNPs). Accurate comprehension of the impact of selected SGAs on MetS and its risk factors largely depends on information pertaining to drug treatments. SNPs are valuable because they have the potential to influence individual susceptibility to MetS. Importantly, subsequent logistic models will be adjusted for drug treatments and SNPs. Instead of forming the foundation for a research hypothesis endpoint, these variables will be integrated into the modelling process to manage potential confounding effects. This strategy permits researchers to investigate the correlation between MetS and other factors while considering the impact of drug treatments and genetic variations.

### Statistical consideration: Study 3

This investigation will adopt a comprehensive approach by collating clinical and biological data collected at two distinct periods (T0 and T3) to provide a detailed account of both MetS+ and MetS- patients. Collecting data at both T0 and T3 has the advantage of not only obtaining a momentary view of the patients’ health status but also allowing for the study of possible changes over time. For the third study, we sought to explore how PA patterns, dietary habits, eating behaviour, and mood intersect in individuals categorized into three groups: MetS+, MetS-, and a control group. To carry out the analysis of PA based on accelerometers, ActiLife software from ActiGraph will be employed for processing; then, the individual subject data will be further processed utilizing the R package GGIR [48]. The distribution of PA intensity will be explored by aggregating data at the sample level into 60-second intervals. To classify the intensity of physical activity, we will rely on a categorization system based on the scheme proposed by Hildebrand in 2014 [49]. This system classifies activities into categories such as sedentary, light, moderate, and vigorous PA. The thorough analytical procedures undertaken will enable us to clarify and measure distinctions in PA among the three study groups in terms of pattern and intensity. The planned sample size (3 groups with 150 subjects in each) will allow us to detect significant standardized differences among variations (slopes) from T0 to T3 in continuous outcomes of 0.2 (effect size). Calculations were performed assuming a generalized estimating equation (GEE) model assuming a correlation pattern defined by a compound symmetry matrix with a base parameter set to 0.5, a significance level of 0.05 and a power of at least 80%.

## Participant retention strategies

To increase response and participation in the research study conducted by the RISKMet Consortium, all participants will receive a € 30 grocery voucher as overall compensation for their involvement in the ActiGraph study and their visits.

## Quality management and storage

To maintain data quality, we will implement the following protocols: (i) data collection protocols with standardized methods and training; (ii) data validation for accuracy; (iii) data cleaning for reliability; (iv) quality control to address issues related to data compatibility with predefined parameters; (v) data monitoring for protocol compliance; and (vi) data auditing to confirm accuracy and adherence. IRCCS Fatebenefratelli and local sites will store study records in accordance with regulatory requirements, with restricted access for authorized personnel.

### Data access and quality assurance

Partner institutions adhere to local research ethics, national, and EU laws for person-identifiable data. Personal data are collected, shared, and safeguarded to ensure confidentiality. The RISKMet study team, experienced in privacy and safety, keeps participant information confidential and accessible only to authorized personnel for audits. After the project, data sets will be available per ‘IRCCS Fatebenefratelli Data Management Policy’, following legal, ethical, and good practice guidelines for data registration, storage, accessibility, and disposal.

### Data transfer

All transfers of study data are informed by and comply with the European Parliament and the Council of Europe’s Directive 95/46/EC on the protection of individuals concerning the handling of personal data and on the free flow of such information between EU countries. To ensure the security and integrity of data during such transfer, an appropriate documented standard procedure has been established and will be followed without exception. Any study data that are to be transferred between research sites are anonymized before transfer.

### Ethics and dissemination

This project has been approved by ethical committees at all participating centres. The RISKMet consortium, comprising two IRCCS and two NHS sites, embodies balanced partnership merging expertise in mental health, endocrinology, epidemiology, biostatistics, and public health. This consortium features four accomplished principal investigators with complementary skills, boasting a history of research and adept communication of findings. Their combined proficiency enables the consortium to execute the project, enhancing health outcomes for individuals on AP medications. Their experience, collaborative spirit, and effective dissemination of research findings align with the consortium’s mission to improve the health outcomes of such individuals.

The primary focus of RISKMet lies in fostering the active engagement and dedication of healthcare providers while also nurturing the participation and empowerment of patients within their healthcare journeys. The outcome of this project will be a set of recommendations that faithfully capture the perspectives and real-life experiences of patients, their caregivers, and healthcare professionals who play a pivotal role in the care of individuals with BD, SSD and NND. These valuable insights will be widely shared through conferences and rigorously reviewed publications within the professional community. To ensure the transparency and replicability of our research, RISKMet will consistently uphold an open-access policy for all project outcomes. Our approach to data dissemination will adhere strictly to privacy and data protection regulations, along with a commitment to upholding other pertinent ethical considerations.

## Status of the study

Recruitment has not yet started.

### Patients’ involvement

Patient involvement is essential for the success of the RISKMet programme, which seeks to comprehend MetS in individuals taking SGAs. We will establish a patient advisory board that will be asked to provide feedback on the overall study design and on the conduct of the study. A thorough case‒control study actively involves patients in the research process, with their participation being critical to the project’s success. Patient participation extends to various areas, such as sharing their health history and lifestyle habits, undergoing physical examinations, and providing valuable biological samples. This patient-centred approach guarantees that the study’s results are both scientifically rigorous and accurately reflect the real-life experiences and needs of those impacted by MetS and SGA treatment. The RISKMet project aims to enhance both mental and physical health, emphasizing the necessity of involving patients in deepening our comprehension of this multifaceted condition within the discipline of psychiatry.

## Discussion

This study encompasses three interconnected research phases, each targeting different aspects of MetS to provide a holistic understanding of its aetiology.

## Multifaceted objectives

The objectives of the RISKMet project are multipronged, aiming to explore MetS from clinical, biological, and behavioural perspectives. This multifaceted approach is crucial for comprehensively understanding the risk factors, characteristics, and behavioural patterns associated with MetS in AP-treated patients. By focusing on high-risk populations, the project seeks not only to identify risk factors but also to intervene and prevent the progression of this condition, which is a significant contributor to morbidity and mortality in this population.

### Study design and setting

The project is a two-year multicentre, multiphase study that will recruit diverse patient populations from different locations and age groups, including adults and children. This comprehensive approach will span approximately three months with two evaluation time points (T0 and T3) and will involve multiple study sites to enhance robustness and generalizability.

### Characteristics of participants

The study will include individuals with MetS (MetS+), control groups of individuals without MetS (MetS-), and, in the paediatric population, their parents. This comprehensive approach will explore familial genetic susceptibility for MetS and consider potential confounding factors by recruiting healthy controls, thereby enabling investigation of horizontal familial liability for MetS.

### Measures, procedures, and variables

A range of assessments, both clinical and biological, will be conducted to understand various aspects of participants’ well-being, functional status, quality of life, and comorbidities. These assessments are consistent with the DSM-5 criteria. Furthermore, the collection of biological samples, including genomic DNA and RNA, allows for in-depth genetic and transcript analysis, enabling the identification of genetic components related to MetS.

## Significance and innovation

MetS is a complex, multifactorial condition that poses a significant challenge to healthcare professionals due to its uncertain aetiopathogenesis and lack of measurable markers. However, recent advancements have created new opportunities to improve the detection and monitoring of MetS and ensure the correct and safe utilization of SGA medication. One of the crucial advancements in this field is the application of rigorous digital phenotyping on SGA-treated patients, surpassing conventional paper-and-pencil data collection techniques. This leads to the recognition of biochemical indicators that recognize both MetS parameters and the physical alterations instigated by SGA that are associated with metabolic ailments, thus facilitating in-depth comprehension of the fundamental mechanisms behind MetS development. Furthermore, an evaluation of the familial risks associated with MetS and the use of healthy control individuals who possess the same genetic background as the patients can furnish significant insights into the environmental and genetic factors that contribute to the ailment. Expanding the research scope and implementing a variety of innovative techniques will enable healthcare professionals to recognize effective interventions for enhancing the psychological and physical health of individuals undergoing SGA treatment and minimize the likelihood of correlated comorbidities. In general, these advancements possess the potential to transform the methods we utilize to diagnose, monitor, and manage MetS, thus enhancing patient outcomes and alleviating the burden on the global healthcare system.

### Limitations

The primary limitation is the small sample size due to financial constraints and a two-year grant. We have prioritized internal validity with comprehensive assessments, but this study will have limited external validity. The assessment process is time-intensive and may result in higher rejection rates, which can be reduced through strong partnerships with healthcare professionals and patients.

### Conclusions

Several pharmacoepidemiologic studies have shown a significant rise in the use of APs in different clinical populations; this trend has seen a remarkable enhancement since the introduction of SGA. While it is widely recognized that these new compounds have improved the treatment of several clinical conditions, SGA use has been linked to various detrimental health outcomes, including MetS and other physical health complications. Consequently, there is growing attention being paid to devising preventive measures that reduce the risk of comorbidities associated with SGA usage. Another important advantage of providing targeted treatment with SGA is the establishment of a robust evidence base for programs designed to enhance the physical wellbeing of individuals undergoing SGA treatment. Such evidence can aid healthcare experts in identifying effective interventions to mitigate the risk of comorbidities associated with SGA usage. This study will provide helpful information to improve the clinical use of SGA, modifying the risk/benefit ratio in the direction of more benefits and fewer risks.

## Supporting information

S1 Table List of participating and recruiting centres. (PDF)

S2 Table Physical examination essential checklist. (PDF)

S1 File (PDF)

## Abbreviations

APs: Antipsychotics
BD: Bipolar Disorder
CVD: Cardiovascular Disease
ESM: Experience Sampling Method
GPCRs: G Protein-coupled receptors
MetS: Metabolic Syndrome
NDD: Neurodevelopmental Disorder
PA: Physical Activity
SGAs: Second-generation antipsychotics
SSD: Schizophrenia Spectrum Disorder
