# Supplementary material for: Metabolic Syndrome in people treated with Antipsychotics (RISKMet): A multimethod study protocol investigating genetic, behavioural, and environmental risk factors
Source: PLoS One. 2024 May 1;19(5):e0298161. doi: 10.1371/journal.pone.0298161 (PMC11062525; doi:10.1371/journal.pone.0298161)
Supplement: S1 Table — (PDF) [file pone.0298161.s001.pdf]

TABLE 1S

LIST OF PARTICIPATING AND RECRUITING CENTRES

LOCATION	ROLE IN THE PROJECT	NAME OF BENEFICIARY	SITE DESCRIPTION
**Brescia**	Coordinating site	IRCCS Istituto Centro San Giovanni di Dio, FBF (Brescia)	Recruiting adult population sample.
**Bosisio Parini**	Partner, recruiting	IRCCS Eugenio Medea Bosisio Parini	Recruiting paediatric population sample and analysing biological samples for cDNA and DNA analysis.
**Naples**	Partner, not recruiting	Azienda Ospedaliera Universitaria Policlinico Federico II	Carrying out the analyses of biomarker panel and of gut microbiota.
**Palermo**	Partner, recruiting	Azienda Ospedaliera Universitaria Policlinico Paolo Giaccone	Recruiting adult population sample.
