# Supplementary material for: Metabolic Syndrome in people treated with Antipsychotics (RISKMet): A multimethod study protocol investigating genetic, behavioural, and environmental risk factors
Source: PLoS One. 2024 May 1;19(5):e0298161. doi: 10.1371/journal.pone.0298161 (PMC11062525; doi:10.1371/journal.pone.0298161)
Supplement: S2 Table — (PDF) [file pone.0298161.s002.pdf]

TABLE 2S

PHYSICAL EXAMINATION ESSENTIAL CHECKLIST

**RESPONSE OPTIONS**	**YE S**	**N O**	**PARTIA L**
VITAL SIGNS			
**1. Measured pulse rate by palpating radial pulse or auscultated at apex of heart at least 30 sec**			
• **Measured blood pressure in one arm, 2 steps for initial measurement (not baseline record)**<br>1. Use appropriate size cuff,<br>2. placed an inch (2cm) above antecubital space,<br>3. inflated cuff 30mmHg above pulse disappearance (palpate or auscultate),<br>4. deflated cuff at 2-3mm Hg per sec, until 20-30 below the last sound			
• **Measured blood pressure in one arm, 1 step for repeat measurement**<br>1. Use appropriate size cuff,<br>2. placed an inch (2cm) above antecubital space,<br>3. inflated cuff 30mmHg above pulse disappearance (palpate or auscultate),<br>4. deflated cuff at 2-3mm Hg per sec, until 20-30 below the last sound			
• **Measured Respiratory Rate- at least 30 sec**			
NECK			
• **Palpated carotids**<br>1. at the level of thyroid cartilage<br>2. right and left (not same time)			
• **Auscultates Carotids**<br>1. with bell<br>2. bilaterally<br>3. with the patient's breath held			
CHEST AND PULMONARY EXAM			
• **Inspects- chest wall for shape and symmetry**			
• **Performed percussion of posterior lung fields**<br>1. cephalad to caudal (top to bottom)<br>2. bilaterally<br>3. at least three areas (upper lobe to lower lobe)			

• **Performed auscultation of anterior lung fields bilaterally**<br>1. upright, seated<br>2. at two levels, at least (upper lobe and lower)<br>3. both right and left			
• **Performed auscultation of posterior lung fields bilaterally**<br>1. upright, seated<br>2. at least three areas (upper lobe to lower lobe)<br>3. both right and left			
• **Auscultates lateral lung fields -one area each, right and left**			
CARDIAC EXAM			
• **Drapes**<br>1. must be able to listen to skin<br>2. attends to patient comfort, through draping			
• **Inspects**<br>1. precordium<br>2. neck veins, carotid pulse<br>3. apex of the heart (Left Lower Sternal Border, 5th InterCostalSpace)			
• **Palpates heart**<br>1. at apex<br>2. over right ventricle (Left Lower Sternal Border or epigastric area)<br>3. at the base (Right Upper Sternal border, Left Upper Sternal Border)			
• **Auscultates with the patient in 3 positions, i.e.**<br>1. upright, seated<br>2. supine<br>3. left lateral			
• **Auscultates in correct locations (all 4 areas of the heart), i.e.**<br>1. Upper right sternal border (aortic area)<br>2. Upper left sternal border (pulmonic area)<br>3. Lower left sternal border (right ventricular area)<br>4. Apical Impulse (Left ventricular area)			
• **Auscultates with both bell and diaphragm (all 4 areas)**			
EXTREMITY EXAM			
• **Palpates legs for oedema with moderate pressure for 5 seconds**			
PERIPHERAL VASCULAR EXAM			
• **Palpates radial pulses, bilaterally**			

• **Palpates brachial pulses, bilaterally**			
• **Palpates posterior tibial pulses**			
• **Palpates dorsalis pedis pulses**			

Contributions for this checklist from Kim Tartaglia, Jane Goleman, Cami Curren, Paul Weber, Alan Letson, Julie Bishop, Adam Quick, Mary Beth Fontana, Troy Schaffernocker, Udi Nori, Sheryl Pfeil, Maria Lucarelli.
