# Supplementary material for: Metabolic Syndrome in people treated with Antipsychotics (RISKMet): A multimethod study protocol investigating genetic, behavioural, and environmental risk factors
Source: PLoS One. 2024 May 1;19(5):e0298161. doi: 10.1371/journal.pone.0298161 (PMC11062525; doi:10.1371/journal.pone.0298161)
Supplement: S1 File — (PDF) [file pone.0298161.s003.pdf]

Marta Magno

Da: Ricerca Sanitaria <ricercasanitaria@sanita.it>
Inviato: mercoledì 14 dicembre 2022 16:33
A: giovannidegirolamo3@gmail.com
Oggetto: Comunicazione invio convenzioni regolatorie progetti PNRR al Destinatario Istituzionale

A DE GIROLAMO GIOVANNI (email: giovannidegirolamo3@gmail.com)

In qualità di Principal Investigator del proposta progettuale codice PNRR-MAD-2022-12375751 dal titolo "METABOLIC SYNDROME IN PEOPLE TREATED WITH ANTIPSYCHOTICS: A MULTIMETHOD INVESTIGATION OF GENETIC, BEHAVIOURAL AND ENVIRONMENTAL RISK FACTORS (RISKMet)" presentato nell'ambito del bando PNRR, bandito da questo Ministero, con il destinatario Istituzionale FATEBENEFRATELLI si informa la SV che la scrivente direzione generale ha inviato al destinatario istituzionale la convezione regolatoria del progetto in questione che la SV dovrà sottoscrivere entro il 31 dicembre 2022.

Si prega di rivolgersi al Suo destinatario istituzionale per le procedure di sottoscrizione della convenzione e si segnala la necessità di una attenta lettura del testo convenzionale da sottoscrivere.

Direzione Generale della Ricerca ed Innovazione in Sanità

Ministero della Salute
